# Supplementary material for: Modeling glioblastoma heterogeneity as a dynamic network of cell states
Source: Mol Syst Biol. 2021 Sep 16;17(9):e10105. doi: 10.15252/msb.202010105 (PMC8444284; doi:10.15252/msb.202010105)
Supplement: Supplementary file 5 — Source Data for Figure 3 [file MSB-17-e10105-s001.zip › Figure3A_sourcedata/GSEA_3065/nosigdb_state1.GseaPreranked.1622718409535/RICHARDS_NOWAKOWSKI_SCIENCE_2017_VRG_UPREG.html]

Details for gene set RICHARDS\_NOWAKOWSKI\_SCIENCE\_2017\_VRG\_UPREG[GSEA]

|  || Dataset | state1 |
| Phenotype | NoPhenotypeAvailable |
| Upregulated in class | na\_neg |
| GeneSet | RICHARDS\_NOWAKOWSKI\_SCIENCE\_2017\_VRG\_UPREG |
| Enrichment Score (ES) | -0.5534168 |
| Normalized Enrichment Score (NES) | -2.2737937 |
| Nominal p-value | 0.0 |
| FDR q-value | 0.0 |
| FWER p-Value | 0.0 |
Table: GSEA Results Summary

  

Fig 1: Enrichment plot: RICHARDS\_NOWAKOWSKI\_SCIENCE\_2017\_VRG\_UPREG      
 Profile of the Running ES Score & Positions of GeneSet Members on the Rank Ordered List

  

| PROBE | GENE SYMBOL | GENE\_TITLE | RANK IN GENE LIST | RANK METRIC SCORE | RUNNING ES | CORE ENRICHMENT || 1 | FBXO32 |  |  | 39 | 0.523 | 0.0043 | No |
| 2 | STK17A |  |  | 47 | 0.483 | 0.0114 | No |
| 3 | ANXA2 |  |  | 48 | 0.482 | 0.0191 | No |
| 4 | TPI1 |  |  | 52 | 0.473 | 0.0265 | No |
| 5 | CAPN2 |  |  | 65 | 0.434 | 0.0322 | No |
| 6 | MYL6 |  |  | 68 | 0.420 | 0.0387 | No |
| 7 | PHLDA1 |  |  | 83 | 0.389 | 0.0435 | No |
| 8 | ENO1 |  |  | 99 | 0.373 | 0.0480 | No |
| 9 | DBI |  |  | 110 | 0.352 | 0.0526 | No |
| 10 | LGALS3 |  |  | 117 | 0.348 | 0.0576 | No |
| 11 | TAGLN2 |  |  | 139 | 0.328 | 0.0607 | No |
| 12 | MT2A |  |  | 161 | 0.317 | 0.0636 | No |
| 13 | YBX3 |  |  | 163 | 0.314 | 0.0685 | No |
| 14 | VIM |  |  | 175 | 0.308 | 0.0723 | No |
| 15 | ACTN1 |  |  | 230 | 0.284 | 0.0712 | No |
| 16 | HIGD1A |  |  | 257 | 0.275 | 0.0729 | No |
| 17 | GNG5 |  |  | 276 | 0.269 | 0.0753 | No |
| 18 | NOG |  |  | 381 | 0.231 | 0.0681 | No |
| 19 | JUN |  |  | 387 | 0.229 | 0.0713 | No |
| 20 | PDCD6 |  |  | 412 | 0.223 | 0.0723 | No |
| 21 | CACYBP |  |  | 458 | 0.214 | 0.0710 | No |
| 22 | MYL12A |  |  | 495 | 0.207 | 0.0706 | No |
| 23 | PPA1 |  |  | 498 | 0.206 | 0.0737 | No |
| 24 | RGS16 |  |  | 499 | 0.206 | 0.0770 | No |
| 25 | HSPB1 |  |  | 531 | 0.199 | 0.0770 | No |
| 26 | PPP1CB |  |  | 534 | 0.198 | 0.0799 | No |
| 27 | TFAP2C |  |  | 602 | 0.186 | 0.0759 | No |
| 28 | AKAP12 |  |  | 605 | 0.185 | 0.0786 | No |
| 29 | FDFT1 |  |  | 635 | 0.181 | 0.0785 | No |
| 30 | PALLD |  |  | 646 | 0.179 | 0.0803 | No |
| 31 | SERF2 |  |  | 695 | 0.173 | 0.0781 | No |
| 32 | AXL |  |  | 704 | 0.172 | 0.0800 | No |
| 33 | ANXA5 |  |  | 709 | 0.171 | 0.0823 | No |
| 34 | CNN3 |  |  | 727 | 0.168 | 0.0832 | No |
| 35 | GSTP1 |  |  | 851 | 0.151 | 0.0727 | No |
| 36 | GLO1 |  |  | 855 | 0.151 | 0.0748 | No |
| 37 | FAM114A1 |  |  | 900 | 0.145 | 0.0725 | No |
| 38 | CASP3 |  |  | 923 | 0.142 | 0.0725 | No |
| 39 | CALD1 |  |  | 968 | 0.137 | 0.0701 | No |
| 40 | IQGAP2 |  |  | 987 | 0.135 | 0.0704 | No |
| 41 | HMGN3 |  |  | 1008 | 0.133 | 0.0704 | No |
| 42 | TSPAN5 |  |  | 1041 | 0.129 | 0.0691 | No |
| 43 | BOC |  |  | 1100 | 0.122 | 0.0650 | No |
| 44 | MOXD1 |  |  | 1104 | 0.122 | 0.0666 | No |
| 45 | SPRY2 |  |  | 1171 | 0.115 | 0.0615 | No |
| 46 | MEIS2 |  |  | 1230 | 0.110 | 0.0572 | No |
| 47 | EEF1D |  |  | 1244 | 0.109 | 0.0576 | No |
| 48 | CD99 |  |  | 1301 | 0.103 | 0.0533 | No |
| 49 | CKB |  |  | 1368 | 0.098 | 0.0479 | No |
| 50 | MMAB |  |  | 1383 | 0.097 | 0.0480 | No |
| 51 | RIN2 |  |  | 1450 | 0.092 | 0.0425 | No |
| 52 | FABP5 |  |  | 1470 | 0.091 | 0.0420 | No |
| 53 | PDLIM5 |  |  | 1528 | 0.087 | 0.0374 | No |
| 54 | EZR |  |  | 1549 | 0.086 | 0.0367 | No |
| 55 | PHACTR2 |  |  | 1726 | 0.074 | 0.0193 | No |
| 56 | TBC1D1 |  |  | 1786 | 0.071 | 0.0142 | No |
| 57 | NDUFB5 |  |  | 1905 | 0.065 | 0.0029 | No |
| 58 | ALDOA |  |  | 1936 | 0.063 | 0.0007 | No |
| 59 | SFRP1 |  |  | 1944 | 0.063 | 0.0010 | No |
| 60 | TBL1X |  |  | 2048 | 0.057 | -0.0089 | No |
| 61 | REXO2 |  |  | 2066 | 0.056 | -0.0098 | No |
| 62 | SRI |  |  | 2084 | 0.056 | -0.0107 | No |
| 63 | CYR61 |  |  | 2172 | 0.052 | -0.0190 | No |
| 64 | MSN |  |  | 2180 | 0.052 | -0.0189 | No |
| 65 | EFNB2 |  |  | 2187 | 0.051 | -0.0187 | No |
| 66 | PDCD10 |  |  | 2312 | 0.047 | -0.0311 | No |
| 67 | PPP2CB |  |  | 2344 | 0.045 | -0.0336 | No |
| 68 | SEPT11 |  |  | 2419 | 0.043 | -0.0407 | No |
| 69 | HSPH1 |  |  | 2441 | 0.042 | -0.0423 | No |
| 70 | DUSP10 |  |  | 2450 | 0.041 | -0.0424 | No |
| 71 | LRRC17 |  |  | 2509 | 0.039 | -0.0479 | No |
| 72 | RPS27L |  |  | 2723 | 0.032 | -0.0698 | No |
| 73 | OAT |  |  | 2778 | 0.031 | -0.0750 | No |
| 74 | EIF2AK2 |  |  | 2806 | 0.030 | -0.0774 | No |
| 75 | CARHSP1 |  |  | 2827 | 0.030 | -0.0790 | No |
| 76 | CHP1 |  |  | 2864 | 0.029 | -0.0824 | No |
| 77 | CHMP2A |  |  | 2891 | 0.028 | -0.0846 | No |
| 78 | RAI14 |  |  | 3006 | 0.025 | -0.0963 | No |
| 79 | GNG12 |  |  | 3088 | 0.023 | -0.1044 | No |
| 80 | TLK1 |  |  | 3089 | 0.023 | -0.1041 | No |
| 81 | CRYZ |  |  | 3105 | 0.022 | -0.1053 | No |
| 82 | GULP1 |  |  | 3139 | 0.022 | -0.1084 | No |
| 83 | DARS |  |  | 3142 | 0.022 | -0.1083 | No |
| 84 | GLUD1 |  |  | 3290 | 0.018 | -0.1235 | No |
| 85 | BICC1 |  |  | 3300 | 0.018 | -0.1241 | No |
| 86 | COL27A1 |  |  | 3311 | 0.018 | -0.1249 | No |
| 87 | JUND |  |  | 3315 | 0.018 | -0.1249 | No |
| 88 | KAZN |  |  | 3327 | 0.018 | -0.1258 | No |
| 89 | CDCA7L |  |  | 3390 | 0.016 | -0.1321 | No |
| 90 | MAST4 |  |  | 3414 | 0.016 | -0.1342 | No |
| 91 | JADE1 |  |  | 3438 | 0.015 | -0.1364 | No |
| 92 | YAP1 |  |  | 3463 | 0.015 | -0.1387 | No |
| 93 | TOX |  |  | 3469 | 0.015 | -0.1390 | No |
| 94 | DMRTA2 |  |  | 3477 | 0.014 | -0.1395 | No |
| 95 | EEPD1 |  |  | 3518 | 0.013 | -0.1435 | No |
| 96 | INSIG1 |  |  | 3553 | 0.013 | -0.1469 | No |
| 97 | MLEC |  |  | 3567 | 0.012 | -0.1481 | No |
| 98 | MID1 |  |  | 3642 | 0.011 | -0.1557 | No |
| 99 | FHOD3 |  |  | 3786 | 0.008 | -0.1706 | No |
| 100 | C1orf198 |  |  | 3814 | 0.008 | -0.1733 | No |
| 101 | LSS |  |  | 3836 | 0.007 | -0.1754 | No |
| 102 | STK39 |  |  | 3877 | 0.007 | -0.1795 | No |
| 103 | ETV5 |  |  | 3894 | 0.006 | -0.1811 | No |
| 104 | FGFR2 |  |  | 4004 | 0.004 | -0.1925 | No |
| 105 | LITAF |  |  | 4039 | 0.003 | -0.1961 | No |
| 106 | MAT2B |  |  | 4114 | 0.002 | -0.2038 | No |
| 107 | KLF6 |  |  | 4117 | 0.002 | -0.2040 | No |
| 108 | DMD |  |  | 4140 | 0.002 | -0.2063 | No |
| 109 | CDO1 |  |  | 4212 | 0.001 | -0.2138 | No |
| 110 | MGST3 |  |  | 4220 | 0.000 | -0.2145 | No |
| 111 | STXBP3 |  |  | 4241 | -0.000 | -0.2166 | No |
| 112 | PSAT1 |  |  | 4318 | -0.001 | -0.2246 | No |
| 113 | TMEM65 |  |  | 4325 | -0.001 | -0.2252 | No |
| 114 | TLN2 |  |  | 4328 | -0.002 | -0.2254 | No |
| 115 | FAM219A |  |  | 4363 | -0.002 | -0.2289 | No |
| 116 | WEE1 |  |  | 4385 | -0.003 | -0.2311 | No |
| 117 | BBX |  |  | 4535 | -0.005 | -0.2467 | No |
| 118 | BMP7 |  |  | 4562 | -0.006 | -0.2494 | No |
| 119 | PUS7 |  |  | 4582 | -0.006 | -0.2513 | No |
| 120 | SEL1L3 |  |  | 4651 | -0.007 | -0.2583 | No |
| 121 | ZDHHC2 |  |  | 4692 | -0.007 | -0.2624 | No |
| 122 | TCF7L2 |  |  | 4707 | -0.008 | -0.2638 | No |
| 123 | ATP10D |  |  | 4724 | -0.008 | -0.2654 | No |
| 124 | LDLR |  |  | 4731 | -0.008 | -0.2659 | No |
| 125 | GCA |  |  | 4757 | -0.008 | -0.2684 | No |
| 126 | FBN2 |  |  | 4789 | -0.009 | -0.2715 | No |
| 127 | CLDND1 |  |  | 4823 | -0.009 | -0.2748 | No |
| 128 | CUL3 |  |  | 4869 | -0.010 | -0.2794 | No |
| 129 | SLC12A2 |  |  | 4938 | -0.012 | -0.2864 | No |
| 130 | CSMD2 |  |  | 4978 | -0.012 | -0.2903 | No |
| 131 | RHOJ |  |  | 5068 | -0.014 | -0.2994 | No |
| 132 | REEP3 |  |  | 5071 | -0.014 | -0.2994 | No |
| 133 | DOK5 |  |  | 5166 | -0.015 | -0.3091 | No |
| 134 | ALDH9A1 |  |  | 5173 | -0.015 | -0.3095 | No |
| 135 | SLC35D1 |  |  | 5178 | -0.015 | -0.3097 | No |
| 136 | LRIG3 |  |  | 5248 | -0.016 | -0.3167 | No |
| 137 | ARHGAP5 |  |  | 5327 | -0.017 | -0.3246 | No |
| 138 | ZCCHC3 |  |  | 5440 | -0.019 | -0.3361 | No |
| 139 | PDGFC |  |  | 5451 | -0.019 | -0.3369 | No |
| 140 | TP53 |  |  | 5557 | -0.021 | -0.3476 | No |
| 141 | GPAM |  |  | 5568 | -0.021 | -0.3483 | No |
| 142 | TLE4 |  |  | 5578 | -0.021 | -0.3489 | No |
| 143 | PCGF5 |  |  | 5585 | -0.022 | -0.3492 | No |
| 144 | SPATA13 |  |  | 5810 | -0.025 | -0.3724 | No |
| 145 | DHCR24 |  |  | 5843 | -0.026 | -0.3753 | No |
| 146 | LIPG |  |  | 5846 | -0.026 | -0.3751 | No |
| 147 | STK17B |  |  | 5886 | -0.027 | -0.3788 | No |
| 148 | CASC10 |  |  | 5887 | -0.027 | -0.3784 | No |
| 149 | HSPA4L |  |  | 5938 | -0.028 | -0.3832 | No |
| 150 | DTNA |  |  | 6001 | -0.029 | -0.3892 | No |
| 151 | CNTNAP3B |  |  | 6078 | -0.030 | -0.3968 | No |
| 152 | SHROOM3 |  |  | 6100 | -0.031 | -0.3985 | No |
| 153 | ABCD3 |  |  | 6145 | -0.032 | -0.4026 | No |
| 154 | IDI1 |  |  | 6154 | -0.032 | -0.4029 | No |
| 155 | ARL6IP5 |  |  | 6216 | -0.033 | -0.4088 | No |
| 156 | FUT8 |  |  | 6330 | -0.035 | -0.4202 | No |
| 157 | ATP2B4 |  |  | 6357 | -0.036 | -0.4223 | No |
| 158 | TGIF1 |  |  | 6360 | -0.036 | -0.4220 | No |
| 159 | MLLT10 |  |  | 6517 | -0.039 | -0.4378 | No |
| 160 | SCHIP1 |  |  | 6575 | -0.040 | -0.4431 | No |
| 161 | FERMT2 |  |  | 6579 | -0.040 | -0.4428 | No |
| 162 | TOB2 |  |  | 6650 | -0.042 | -0.4495 | No |
| 163 | RGL1 |  |  | 6690 | -0.043 | -0.4529 | No |
| 164 | DENND1A |  |  | 6738 | -0.044 | -0.4572 | No |
| 165 | NBPF14 |  |  | 6792 | -0.045 | -0.4620 | No |
| 166 | PEA15 |  |  | 6830 | -0.046 | -0.4652 | No |
| 167 | AEBP2 |  |  | 6892 | -0.047 | -0.4709 | No |
| 168 | ING3 |  |  | 6942 | -0.048 | -0.4753 | No |
| 169 | DDAH1 |  |  | 6943 | -0.049 | -0.4745 | No |
| 170 | CTDSP2 |  |  | 6970 | -0.049 | -0.4764 | No |
| 171 | PPM1K |  |  | 6972 | -0.049 | -0.4757 | No |
| 172 | EMC2 |  |  | 7044 | -0.051 | -0.4824 | No |
| 173 | CTBP2 |  |  | 7102 | -0.053 | -0.4876 | No |
| 174 | ZDHHC13 |  |  | 7153 | -0.054 | -0.4919 | No |
| 175 | ZC3HAV1 |  |  | 7178 | -0.055 | -0.4936 | No |
| 176 | TAOK2 |  |  | 7231 | -0.056 | -0.4982 | No |
| 177 | RAB31 |  |  | 7256 | -0.057 | -0.4998 | No |
| 178 | REST |  |  | 7263 | -0.057 | -0.4995 | No |
| 179 | IQSEC1 |  |  | 7311 | -0.058 | -0.5035 | No |
| 180 | ALDH6A1 |  |  | 7321 | -0.059 | -0.5035 | No |
| 181 | ITGA6 |  |  | 7339 | -0.059 | -0.5044 | No |
| 182 | STX16 |  |  | 7367 | -0.060 | -0.5062 | No |
| 183 | LARP7 |  |  | 7371 | -0.060 | -0.5056 | No |
| 184 | RALGAPA2 |  |  | 7425 | -0.061 | -0.5102 | No |
| 185 | PDCD4 |  |  | 7452 | -0.062 | -0.5119 | No |
| 186 | GLI3 |  |  | 7475 | -0.062 | -0.5132 | No |
| 187 | SEPT3 |  |  | 7486 | -0.063 | -0.5133 | No |
| 188 | DOCK1 |  |  | 7505 | -0.064 | -0.5142 | No |
| 189 | CNTLN |  |  | 7518 | -0.064 | -0.5144 | No |
| 190 | ACBD5 |  |  | 7533 | -0.064 | -0.5148 | No |
| 191 | NFE2L2 |  |  | 7575 | -0.066 | -0.5181 | No |
| 192 | NADK2 |  |  | 7596 | -0.066 | -0.5191 | No |
| 193 | STAT3 |  |  | 7597 | -0.066 | -0.5181 | No |
| 194 | SPAG16 |  |  | 7619 | -0.067 | -0.5192 | No |
| 195 | CELSR1 |  |  | 7680 | -0.069 | -0.5244 | No |
| 196 | SAMD4A |  |  | 7719 | -0.071 | -0.5273 | No |
| 197 | VCL |  |  | 7729 | -0.071 | -0.5271 | No |
| 198 | HEG1 |  |  | 7742 | -0.071 | -0.5272 | No |
| 199 | SPRED1 |  |  | 7743 | -0.071 | -0.5260 | No |
| 200 | MAPK1 |  |  | 7779 | -0.072 | -0.5286 | No |
| 201 | MEIS1 |  |  | 7794 | -0.073 | -0.5289 | No |
| 202 | H6PD |  |  | 7809 | -0.074 | -0.5292 | No |
| 203 | HOPX |  |  | 7850 | -0.075 | -0.5322 | No |
| 204 | SCRN1 |  |  | 7886 | -0.077 | -0.5346 | No |
| 205 | IRF2BP2 |  |  | 7887 | -0.077 | -0.5334 | No |
| 206 | OTUD7B |  |  | 7889 | -0.077 | -0.5322 | No |
| 207 | ADAMTS6 |  |  | 7912 | -0.078 | -0.5333 | No |
| 208 | HEATR5A |  |  | 7916 | -0.078 | -0.5324 | No |
| 209 | LAMA1 |  |  | 7949 | -0.079 | -0.5344 | No |
| 210 | BARD1 |  |  | 7980 | -0.080 | -0.5363 | No |
| 211 | SEPT9 |  |  | 7984 | -0.081 | -0.5353 | No |
| 212 | SOAT1 |  |  | 8031 | -0.082 | -0.5389 | No |
| 213 | SMAD5 |  |  | 8035 | -0.083 | -0.5378 | No |
| 214 | MSI1 |  |  | 8064 | -0.084 | -0.5394 | No |
| 215 | CTNND1 |  |  | 8094 | -0.085 | -0.5411 | No |
| 216 | EVI5 |  |  | 8095 | -0.085 | -0.5398 | No |
| 217 | ELOVL5 |  |  | 8115 | -0.086 | -0.5404 | No |
| 218 | ARHGEF40 |  |  | 8170 | -0.089 | -0.5446 | No |
| 219 | LRP4 |  |  | 8181 | -0.089 | -0.5443 | No |
| 220 | RAB3GAP2 |  |  | 8198 | -0.089 | -0.5445 | No |
| 221 | NPHP3 |  |  | 8200 | -0.089 | -0.5432 | No |
| 222 | PHIP |  |  | 8201 | -0.089 | -0.5417 | No |
| 223 | PTGFRN |  |  | 8213 | -0.090 | -0.5414 | No |
| 224 | NUMA1 |  |  | 8218 | -0.090 | -0.5404 | No |
| 225 | HSDL2 |  |  | 8225 | -0.090 | -0.5396 | No |
| 226 | LPP |  |  | 8255 | -0.092 | -0.5412 | No |
| 227 | PDLIM3 |  |  | 8278 | -0.093 | -0.5420 | No |
| 228 | NCKAP5 |  |  | 8280 | -0.094 | -0.5406 | No |
| 229 | CA12 |  |  | 8291 | -0.094 | -0.5401 | No |
| 230 | IFI6 |  |  | 8316 | -0.095 | -0.5411 | No |
| 231 | PCMTD1 |  |  | 8328 | -0.095 | -0.5407 | No |
| 232 | PREX1 |  |  | 8406 | -0.099 | -0.5472 | No |
| 233 | FOXK1 |  |  | 8407 | -0.099 | -0.5456 | No |
| 234 | INTU |  |  | 8410 | -0.100 | -0.5442 | No |
| 235 | PTPRF |  |  | 8439 | -0.101 | -0.5455 | No |
| 236 | GRB14 |  |  | 8474 | -0.103 | -0.5475 | No |
| 237 | AKAP13 |  |  | 8523 | -0.107 | -0.5508 | No |
| 238 | TMX1 |  |  | 8524 | -0.107 | -0.5491 | No |
| 239 | RYK |  |  | 8550 | -0.108 | -0.5500 | No |
| 240 | RCN1 |  |  | 8553 | -0.108 | -0.5484 | No |
| 241 | UBXN4 |  |  | 8571 | -0.110 | -0.5485 | No |
| 242 | RDX |  |  | 8619 | -0.112 | -0.5516 | Yes |
| 243 | GPC4 |  |  | 8624 | -0.113 | -0.5502 | Yes |
| 244 | SH3BGRL |  |  | 8627 | -0.113 | -0.5486 | Yes |
| 245 | STAG2 |  |  | 8629 | -0.113 | -0.5469 | Yes |
| 246 | DAD1 |  |  | 8655 | -0.114 | -0.5477 | Yes |
| 247 | COL11A1 |  |  | 8666 | -0.115 | -0.5469 | Yes |
| 248 | SRGAP2C |  |  | 8682 | -0.116 | -0.5466 | Yes |
| 249 | CTNNA1 |  |  | 8686 | -0.116 | -0.5450 | Yes |
| 250 | SGSM2 |  |  | 8709 | -0.118 | -0.5454 | Yes |
| 251 | AIF1L |  |  | 8722 | -0.119 | -0.5448 | Yes |
| 252 | NAPEPLD |  |  | 8761 | -0.122 | -0.5468 | Yes |
| 253 | MYO6 |  |  | 8788 | -0.124 | -0.5476 | Yes |
| 254 | ZFP36L1 |  |  | 8789 | -0.124 | -0.5456 | Yes |
| 255 | SDCBP |  |  | 8790 | -0.124 | -0.5436 | Yes |
| 256 | GTF2H2 |  |  | 8791 | -0.124 | -0.5416 | Yes |
| 257 | EGR1 |  |  | 8797 | -0.124 | -0.5401 | Yes |
| 258 | TRIB2 |  |  | 8853 | -0.129 | -0.5438 | Yes |
| 259 | ID4 |  |  | 8856 | -0.129 | -0.5419 | Yes |
| 260 | TMEM170A |  |  | 8873 | -0.131 | -0.5415 | Yes |
| 261 | ARAP2 |  |  | 8907 | -0.134 | -0.5428 | Yes |
| 262 | ATXN2 |  |  | 8926 | -0.136 | -0.5425 | Yes |
| 263 | NTRK3 |  |  | 8930 | -0.137 | -0.5406 | Yes |
| 264 | PHYHIPL |  |  | 8937 | -0.137 | -0.5390 | Yes |
| 265 | ZMAT1 |  |  | 8947 | -0.138 | -0.5377 | Yes |
| 266 | FAM19A5 |  |  | 8956 | -0.139 | -0.5363 | Yes |
| 267 | SHC3 |  |  | 8972 | -0.141 | -0.5357 | Yes |
| 268 | FSTL1 |  |  | 8976 | -0.141 | -0.5337 | Yes |
| 269 | FLCN |  |  | 8981 | -0.141 | -0.5318 | Yes |
| 270 | RBM39 |  |  | 8983 | -0.141 | -0.5297 | Yes |
| 271 | TSPAN6 |  |  | 8985 | -0.142 | -0.5275 | Yes |
| 272 | RHOQ |  |  | 9019 | -0.144 | -0.5287 | Yes |
| 273 | GATM |  |  | 9049 | -0.147 | -0.5293 | Yes |
| 274 | PTAR1 |  |  | 9056 | -0.148 | -0.5276 | Yes |
| 275 | SLC25A37 |  |  | 9067 | -0.149 | -0.5262 | Yes |
| 276 | LPIN2 |  |  | 9075 | -0.150 | -0.5246 | Yes |
| 277 | SALL1 |  |  | 9084 | -0.151 | -0.5230 | Yes |
| 278 | GAB1 |  |  | 9132 | -0.156 | -0.5254 | Yes |
| 279 | PLCE1 |  |  | 9140 | -0.157 | -0.5236 | Yes |
| 280 | LHX2 |  |  | 9142 | -0.157 | -0.5212 | Yes |
| 281 | VCAN |  |  | 9151 | -0.158 | -0.5195 | Yes |
| 282 | FGFR1 |  |  | 9157 | -0.159 | -0.5174 | Yes |
| 283 | FAM171B |  |  | 9158 | -0.160 | -0.5149 | Yes |
| 284 | ZFP36L2 |  |  | 9162 | -0.160 | -0.5126 | Yes |
| 285 | NPAS3 |  |  | 9181 | -0.163 | -0.5119 | Yes |
| 286 | SYNE1 |  |  | 9206 | -0.166 | -0.5117 | Yes |
| 287 | ROBO1 |  |  | 9208 | -0.167 | -0.5091 | Yes |
| 288 | LIFR |  |  | 9216 | -0.168 | -0.5072 | Yes |
| 289 | DNAJC1 |  |  | 9228 | -0.169 | -0.5056 | Yes |
| 290 | SCAF11 |  |  | 9232 | -0.170 | -0.5032 | Yes |
| 291 | AASS |  |  | 9237 | -0.171 | -0.5008 | Yes |
| 292 | SEPT2 |  |  | 9246 | -0.172 | -0.4989 | Yes |
| 293 | LRIG1 |  |  | 9256 | -0.173 | -0.4971 | Yes |
| 294 | SNX13 |  |  | 9270 | -0.175 | -0.4956 | Yes |
| 295 | LPCAT1 |  |  | 9274 | -0.175 | -0.4931 | Yes |
| 296 | NOTCH2 |  |  | 9279 | -0.177 | -0.4907 | Yes |
| 297 | HES1 |  |  | 9280 | -0.177 | -0.4878 | Yes |
| 298 | PDPN |  |  | 9284 | -0.177 | -0.4853 | Yes |
| 299 | CTNNB1 |  |  | 9294 | -0.179 | -0.4833 | Yes |
| 300 | SIPA1L1 |  |  | 9296 | -0.180 | -0.4806 | Yes |
| 301 | FAT1 |  |  | 9301 | -0.181 | -0.4781 | Yes |
| 302 | PAG1 |  |  | 9322 | -0.185 | -0.4772 | Yes |
| 303 | ZFHX4 |  |  | 9346 | -0.189 | -0.4766 | Yes |
| 304 | NRBP2 |  |  | 9350 | -0.190 | -0.4738 | Yes |
| 305 | AGO3 |  |  | 9352 | -0.190 | -0.4709 | Yes |
| 306 | MEGF10 |  |  | 9353 | -0.190 | -0.4678 | Yes |
| 307 | SYNE2 |  |  | 9362 | -0.193 | -0.4655 | Yes |
| 308 | PTPRG |  |  | 9370 | -0.194 | -0.4631 | Yes |
| 309 | TRPS1 |  |  | 9385 | -0.198 | -0.4614 | Yes |
| 310 | MSMO1 |  |  | 9391 | -0.199 | -0.4587 | Yes |
| 311 | RFTN2 |  |  | 9402 | -0.200 | -0.4566 | Yes |
| 312 | BICD1 |  |  | 9419 | -0.205 | -0.4549 | Yes |
| 313 | TFPI |  |  | 9427 | -0.206 | -0.4524 | Yes |
| 314 | ATP1B2 |  |  | 9441 | -0.210 | -0.4503 | Yes |
| 315 | FKBP9 |  |  | 9445 | -0.211 | -0.4472 | Yes |
| 316 | TTYH3 |  |  | 9453 | -0.214 | -0.4445 | Yes |
| 317 | FKBP10 |  |  | 9455 | -0.215 | -0.4412 | Yes |
| 318 | PHLPP1 |  |  | 9463 | -0.217 | -0.4384 | Yes |
| 319 | JAM3 |  |  | 9485 | -0.222 | -0.4370 | Yes |
| 320 | SPAG9 |  |  | 9487 | -0.223 | -0.4336 | Yes |
| 321 | ZEB1 |  |  | 9492 | -0.224 | -0.4304 | Yes |
| 322 | MSI2 |  |  | 9498 | -0.225 | -0.4273 | Yes |
| 323 | NOTCH3 |  |  | 9501 | -0.226 | -0.4238 | Yes |
| 324 | SLC16A1 |  |  | 9509 | -0.227 | -0.4209 | Yes |
| 325 | TRIM9 |  |  | 9512 | -0.229 | -0.4174 | Yes |
| 326 | DAG1 |  |  | 9514 | -0.229 | -0.4138 | Yes |
| 327 | HTRA1 |  |  | 9515 | -0.229 | -0.4101 | Yes |
| 328 | IL6ST |  |  | 9519 | -0.231 | -0.4067 | Yes |
| 329 | TMEM98 |  |  | 9527 | -0.233 | -0.4037 | Yes |
| 330 | DKK3 |  |  | 9558 | -0.244 | -0.4029 | Yes |
| 331 | PTPN13 |  |  | 9562 | -0.245 | -0.3993 | Yes |
| 332 | FOS |  |  | 9566 | -0.247 | -0.3956 | Yes |
| 333 | MAGI1 |  |  | 9576 | -0.250 | -0.3926 | Yes |
| 334 | APC |  |  | 9577 | -0.251 | -0.3885 | Yes |
| 335 | CST3 |  |  | 9581 | -0.254 | -0.3847 | Yes |
| 336 | TJP1 |  |  | 9592 | -0.257 | -0.3817 | Yes |
| 337 | TNC |  |  | 9605 | -0.262 | -0.3787 | Yes |
| 338 | JAM2 |  |  | 9612 | -0.263 | -0.3751 | Yes |
| 339 | TMEM132B |  |  | 9613 | -0.264 | -0.3708 | Yes |
| 340 | CREB5 |  |  | 9616 | -0.265 | -0.3668 | Yes |
| 341 | MRC2 |  |  | 9620 | -0.266 | -0.3628 | Yes |
| 342 | NES |  |  | 9626 | -0.270 | -0.3590 | Yes |
| 343 | HMGCR |  |  | 9640 | -0.277 | -0.3559 | Yes |
| 344 | IFITM3 |  |  | 9643 | -0.277 | -0.3516 | Yes |
| 345 | APBB2 |  |  | 9645 | -0.277 | -0.3473 | Yes |
| 346 | ABAT |  |  | 9646 | -0.278 | -0.3428 | Yes |
| 347 | KIF1B |  |  | 9649 | -0.282 | -0.3384 | Yes |
| 348 | ITGAV |  |  | 9650 | -0.282 | -0.3339 | Yes |
| 349 | RFX4 |  |  | 9655 | -0.285 | -0.3297 | Yes |
| 350 | LRP6 |  |  | 9656 | -0.286 | -0.3251 | Yes |
| 351 | SOX2 |  |  | 9680 | -0.298 | -0.3227 | Yes |
| 352 | SRGAP3 |  |  | 9689 | -0.304 | -0.3187 | Yes |
| 353 | SEMA6A |  |  | 9690 | -0.304 | -0.3138 | Yes |
| 354 | EMP2 |  |  | 9696 | -0.310 | -0.3093 | Yes |
| 355 | TSPAN3 |  |  | 9713 | -0.326 | -0.3057 | Yes |
| 356 | SLC35F1 |  |  | 9715 | -0.327 | -0.3006 | Yes |
| 357 | TMBIM6 |  |  | 9716 | -0.328 | -0.2953 | Yes |
| 358 | HMGCS1 |  |  | 9718 | -0.329 | -0.2901 | Yes |
| 359 | MYO10 |  |  | 9725 | -0.335 | -0.2853 | Yes |
| 360 | MLC1 |  |  | 9728 | -0.338 | -0.2801 | Yes |
| 361 | SGCE |  |  | 9733 | -0.341 | -0.2750 | Yes |
| 362 | PSAP |  |  | 9742 | -0.351 | -0.2702 | Yes |
| 363 | ZBTB20 |  |  | 9743 | -0.352 | -0.2645 | Yes |
| 364 | TMED10 |  |  | 9750 | -0.356 | -0.2594 | Yes |
| 365 | FZD3 |  |  | 9753 | -0.357 | -0.2538 | Yes |
| 366 | SOX9 |  |  | 9754 | -0.358 | -0.2481 | Yes |
| 367 | ITM2C |  |  | 9758 | -0.363 | -0.2425 | Yes |
| 368 | SCD |  |  | 9762 | -0.370 | -0.2369 | Yes |
| 369 | ETV1 |  |  | 9764 | -0.371 | -0.2310 | Yes |
| 370 | NOTCH1 |  |  | 9780 | -0.391 | -0.2263 | Yes |
| 371 | SYT11 |  |  | 9785 | -0.399 | -0.2203 | Yes |
| 372 | SQLE |  |  | 9790 | -0.408 | -0.2141 | Yes |
| 373 | ITGB8 |  |  | 9794 | -0.418 | -0.2077 | Yes |
| 374 | CLU |  |  | 9795 | -0.418 | -0.2010 | Yes |
| 375 | QKI |  |  | 9803 | -0.430 | -0.1948 | Yes |
| 376 | CANX |  |  | 9804 | -0.431 | -0.1878 | Yes |
| 377 | SAT1 |  |  | 9807 | -0.445 | -0.1809 | Yes |
| 378 | C1orf61 |  |  | 9813 | -0.479 | -0.1737 | Yes |
| 379 | SEPT7 |  |  | 9821 | -0.500 | -0.1663 | Yes |
| 380 | SLC1A3 |  |  | 9823 | -0.509 | -0.1582 | Yes |
| 381 | FABP7 |  |  | 9825 | -0.513 | -0.1501 | Yes |
| 382 | DDR1 |  |  | 9829 | -0.524 | -0.1419 | Yes |
| 383 | PON2 |  |  | 9835 | -0.554 | -0.1335 | Yes |
| 384 | CCND2 |  |  | 9842 | -0.611 | -0.1243 | Yes |
| 385 | FGFBP3 |  |  | 9850 | -0.651 | -0.1145 | Yes |
| 386 | SEMA5A |  |  | 9860 | -0.749 | -0.1034 | Yes |
| 387 | PTPRZ1 |  |  | 9869 | -0.918 | -0.0895 | Yes |
| 388 | TTYH1 |  |  | 9872 | -1.033 | -0.0730 | Yes |
| 389 | GPM6B |  |  | 9873 | -1.091 | -0.0554 | Yes |
| 390 | PTN |  |  | 9874 | -1.107 | -0.0376 | Yes |
| 391 | GFAP |  |  | 9877 | -1.174 | -0.0188 | Yes |
| 392 | BCAN |  |  | 9878 | -1.175 | 0.0001 | Yes |
Table: GSEA details [plain text format]

  

Fig 2: RICHARDS\_NOWAKOWSKI\_SCIENCE\_2017\_VRG\_UPREG: Random ES distribution      
 Gene set null distribution of ES for **RICHARDS\_NOWAKOWSKI\_SCIENCE\_2017\_VRG\_UPREG**
